# Leveraging natural history biorepositories as a global, decentralized, pathogen surveillance network

**DOI:** 10.1371/journal.ppat.1009583

**Published:** 2021-06-03

**Authors:** Jocelyn P. Colella, John Bates, Santiago F. Burneo, M. Alejandra Camacho, Carlos Carrion Bonilla, Isabel Constable, Guillermo D’Elía, Jonathan L. Dunnum, Stephen Greiman, Eric P. Hoberg, Enrique Lessa, Schuyler W. Liphardt, Manuela Londoño-Gaviria, Elizabeth Losos, Holly L. Lutz, Nicté Ordóñez Garza, A. Townsend Peterson, María Laura Martin, Camila C. Ribas, Bruce Struminger, Fernando Torres-Pérez, Cody W. Thompson, Marcelo Weksler, Joseph A. Cook

**Affiliations:** 1 University of Kansas, Biodiversity Institute, Lawrence, Kansas, United States of America; 2 Field Museum of Natural History, Chicago, Illinois, United States of America; 3 Sección de Mastozoología, Museo de Zoología, Facultad de Ciencias Exactas y Naturales, Pontificia Universidad Católica del Ecuador, Quito, Pichincha, Ecuador; 4 University of New Mexico, Department of Biology and Museum of Southwestern Biology, Albuquerque, New Mexico, United States of America; 5 Museo de Zoologiá, Escuela de Biología, Pontificia Universidad Catolica del Ecuador, Quito, Ecuador; 6 Museum of Southwestern Biology, University of New Mexico, Albuquerque, New Mexico, United States of America; 7 Instituto de Ciencias Ambientales y Evolutivas, Universidad Austral de Chile, Valdivia, Chile; 8 Department of Biology, Georgia Southern University, Statesboro, Georgia, United States of America; 9 University of Wisconsin, School of Veterinary Medicine, Department of Pathobiological Sciences, Madison, Wisconsin, United States of America; 10 Facultad de Ciencias, Universidad de la República, Montevideo, Uruguay; 11 Duke University, Durham, North Carolina, United States of America; 12 University of California San Diego, Scripps Institution of Oceanography, La Jolla, California, United States of America; 13 Museum of Texas Tech University, Lubbock, Texas, United States of America; 14 Instituto Nacional de Biodiversidad-INABIO, Ecuador; 15 Instituto Nacional de Enfermedades Virales Humanas “Dr. Julio I Maiztegui” ANLIS, Argentina; 16 Instituto Nacional de Pesquisas da Amazônia—INPA, Manaus, Amazonas, Brazil; 17 University of New Mexico Health Sciences Center ECHO Institute, Albuquerque, New Mexico, United States of America; 18 Instituto de Biología, Pontificia Universidad Católica de Valparaíso, Valparaíso, Chile; 19 Department of Ecology and Evolutionary Biology and the Museum of Zoology, University of Michigan, Ann Arbor, Michigan, United States of America; 20 Departamento de Vertebrados, Museu Nacional, Universidade Federal do Rio de Janeiro, Rio de Janeiro, Brazil; University of Colorado Denver, UNITED STATES

## Abstract

The Severe Acute Respiratory Syndrome Coronavirus 2 (SARS-CoV-2) pandemic reveals a major gap in global biosecurity infrastructure: a lack of publicly available biological samples representative across space, time, and taxonomic diversity. The shortfall, in this case for vertebrates, prevents accurate and rapid identification and monitoring of emerging pathogens and their reservoir host(s) and precludes extended investigation of ecological, evolutionary, and environmental associations that lead to human infection or spillover. Natural history museum biorepositories form the backbone of a critically needed, decentralized, global network for zoonotic pathogen surveillance, yet this infrastructure remains marginally developed, underutilized, underfunded, and disconnected from public health initiatives. Proactive detection and mitigation for emerging infectious diseases (EIDs) requires expanded biodiversity infrastructure and training (particularly in biodiverse and lower income countries) and new communication pipelines that connect biorepositories and biomedical communities. To this end, we highlight a novel adaptation of Project ECHO’s virtual community of practice model: Museums and Emerging Pathogens in the Americas (MEPA). MEPA is a virtual network aimed at fostering communication, coordination, and collaborative problem-solving among pathogen researchers, public health officials, and biorepositories in the Americas. MEPA now acts as a model of effective international, interdisciplinary collaboration that can and should be replicated in other biodiversity hotspots. We encourage deposition of wildlife specimens and associated data with public biorepositories, regardless of original collection purpose, and urge biorepositories to embrace new specimen sources, types, and uses to maximize strategic growth and utility for EID research. Taxonomically, geographically, and temporally deep biorepository archives serve as the foundation of a proactive and increasingly predictive approach to zoonotic spillover, risk assessment, and threat mitigation.

“We are not students of some subject matter, but students of problems. And problems may cut right across the borders of any subject matter or discipline.”–Karl Popper

## Introduction

The Severe Acute Respiratory Syndrome Coronavirus 2 (SARS-CoV-2) pandemic has revealed critical weaknesses in international biosecurity and pandemic preparedness [1–5]: There are no global wildlife surveillance systems contributing to biorepositories to enable monitoring of emerging zoonotic diseases across space and time and, in consequence, international biorepository capacities are insufficient to permit researchers to identify pathogens and hosts, rapidly and reliably. Development of a global pathogen surveillance and biorepository network would facilitate proactive pandemic preparedness for the first time by enabling early detection, regular monitoring, and the development of an evolutionary framework for spillover prediction [6]. A shift toward a proactive response to emerging infectious diseases (EIDs) would significantly reduce the human and financial costs of new and reemerging diseases [7–10]. Indeed, coordinated research and surveillance efforts to mitigate EID impacts are recognized as an urgent need by the World Health Organization (WHO), Food and Agricultural Organization (FAO), and the World Organization for Animal Health [11]. The good news is that the beginnings of an international, decentralized biorepository network already exist in the form of the “Global Museum,” the geographically dispersed, international community of natural history museums that is increasingly digitally connected [12–13]. Each natural history museum is also a biorepository, dedicated to the long-term preservation of biological materials, including skins, skeletons, cryogenically frozen tissues, and their associated data (e.g., occurrence, pathogen/symbiont, ecological, environmental, etc.). With modest investment and modifications in infrastructure and communication systems, these dispersed nodes could be expanded and connected to form a powerful international system for emerging pathogen surveillance and monitoring [9].

Natural history collections (hereafter, biorepositories) are cumulative records of the biosphere that grow through the accumulation of new specimens over time. For vertebrates, a specimen may consist of a whole organism or any derivative of an organism (e.g., tissues, symbionts [viruses, bacteria, fungi, and eukaryotic parasites], audiovisual data, etc.), with associated metadata on where and when the specimen was collected [14–15]. The physical and digital information stored and curated in biorepositories is precisely the material needed to identify the origin(s), source(s), and environmental associations of zoonotic pathogens and their hosts, which positions biorepositories as a critical component of an updated “One Health” approach to detecting, characterizing, and mitigating effects of emerging zoonoses [3,16–17].

To fulfill their promise as an international system for zoonotic pathogen surveillance and as a foundation for effective public health response, biorepository infrastructure and capacity must be expanded on a global scale to accommodate greater specimen volumes, incoming from disease-related and other wildlife surveys, and outgoing, for use in EID research [18–22]. Expanded biorepository infrastructure is particularly necessary for lower-income and biodiverse countries, which have some of the highest risk and frequency of zoonotic spillover and greatest lag times between disease emergence and identification of wildlife sources [7]. To coordinate global wildlife surveillance across space and time, new international communication pipelines that better unite public health officials, epidemiologists, and local communities with biorepositories must be developed in parallel with infrastructure improvements. Here, we outline next steps for the strategic expansion of international biodiversity infrastructure, to form a decentralized pathogen surveillance network, and propose a new model of international, transdisciplinary communication and collaboration.

### A proactive approach to EIDs is possible through biorepositories

Wildlife specimens housed in museum biorepositories are proven, but underutilized tools for detection and identification of zoonotic pathogens and their wildlife hosts [5,23–28]. Archived tissues were used to track down the zoonotic origins of 1918 Spanish Influenza [29–30] and 1993 outbreaks of Sin Nombre virus in the American Southwest [24,31]. Wildlife sampling of leaf-nosed bats in Ghana recently identified a wild reservoir of rubella, a viral disease whose origins and natural reservoirs had remained unknown for >100 years after emerging in humans [32]. Genetic screening of wildlife in China recently identified horseshoe bats as a potential reservoir for SARS-CoV-2 [33,34]; however, in this case, a paucity of open-access voucher specimens has hindered expanded host screening and limited investigation into the ecology, evolution, and environmental associations of seropositive samples, leaving SARS-CoV-2 emergence a global mystery [4,35–36].

**Fig 1:**
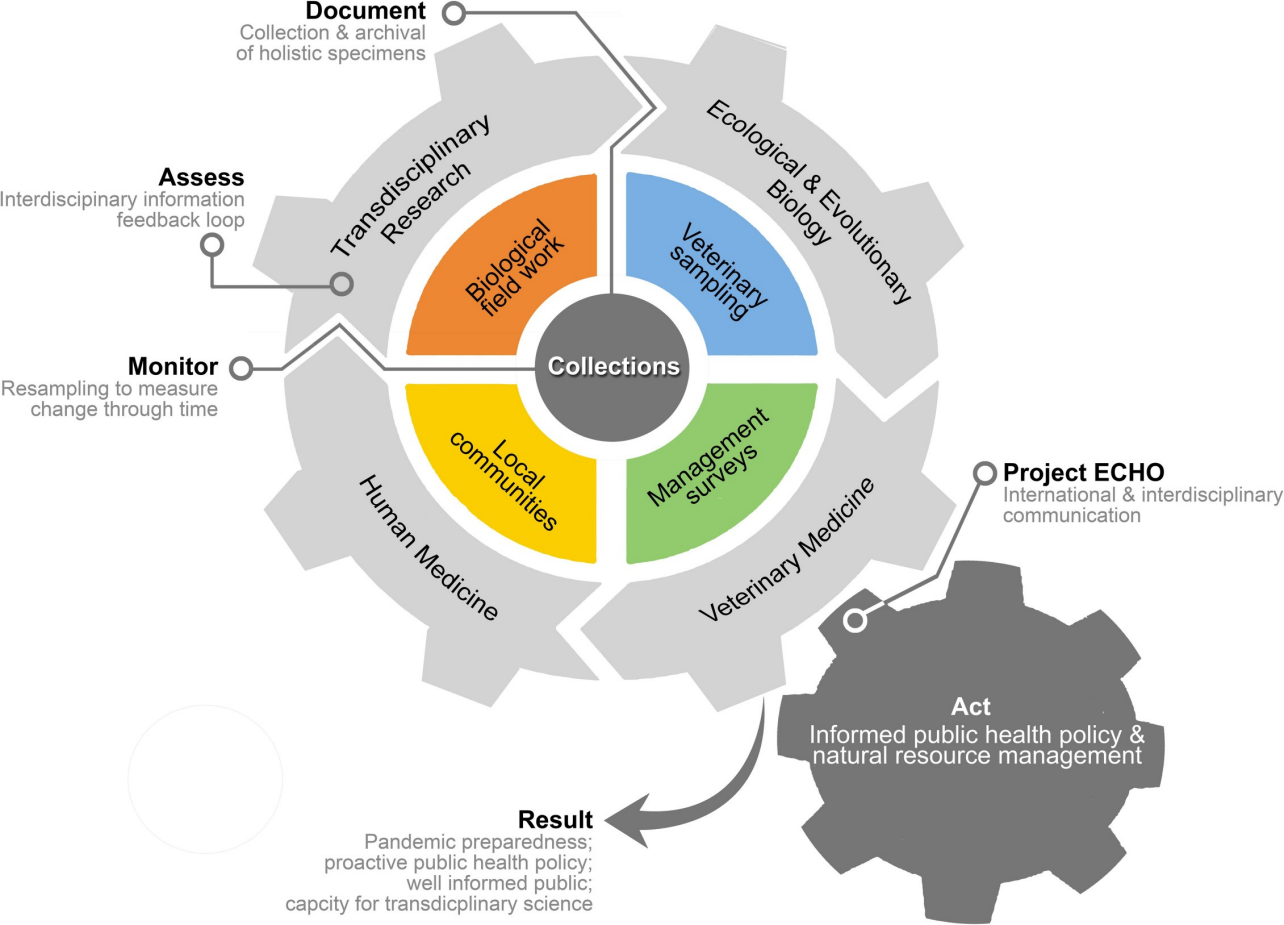
Schematic detailing the central role of biorepositories in fueling EID research and response, based on the DAMA model proposed by Brooks and colleagues [6,49]. To be most effective for EID research and response, multidisciplinary and collaborative specimen sourcing followed by permanent archiving with biorepositories will be necessary to **document** and **assess** baseline conditions of pathogens and hosts. Periodic resampling of established localities is essential to **monitoring** change through time, whereby the availability of both baseline and resampling specimens and data through biorepositories catalyzes transdisciplinary research (e.g., virology, ecology, evolutionary biology, etc.) or **assessment**. Research, in turn, informs both veterinary and human medicine and, when effectively communicated through publications, presentations, and new networks of international and interdisciplinary communication (e.g., Project ECHO), this information drives **action** in the form of public health policy and natural resource management, creating a positive feedback loop that contributes to improved pandemic preparedness, proactive public health policies, and a better informed society. DAMA, Documentation, Assessment, Monitoring, Action; EID, emerging infectious disease; ECHO, Extension for Community Healthcare Outcomes.

Most EIDs in humans are zoonotic in origin, including rabies, yellow fever, salmonella, hantavirus, Ebola, and ringworm, among others, and >60% are transmitted directly from animal hosts [37–38]. Current understanding of global pathogen diversity remains largely incomplete [39–43], with tens of thousands of pathogenic species remaining undiscovered or undescribed [44–46]. Reactive attempts to sample and screen wildlife after pathogen emergence in humans delays threat mitigation [47]. Waiting until a pathogen triggers an epidemic, or worse, a global pandemic is expensive [9,48], does little for future pandemic prevention or preparedness [10,49], and will be diminishingly feasible as human populations become increasingly globally connected [50]. For example, in reaction to the ongoing SARS-CoV-2 pandemic, there are a suite of new research funding opportunities and major strides have been made in viral discovery and spillover prediction; yet, of the nearly 75,000 animals recently surveyed by Grange and colleagues [51] as part of the PREDICT Project [52], not one specimen was permanently archived in a public biorepository, and when there was ambiguity surrounding host identity, only a generic-level name was used. This “business as usual” approach to One Health sampling, excludes biorepositories, their taxonomic expertise, responsible vouchering practices (e.g., long-term sample preservation), and data standardization from disease-motivated wildlife sampling. In contrast, inclusion of biorepositories in EID sampling workflows represents an opportunity to simulate diverse scientific research by documenting and serving specimens and important information surrounding hosts, symbionts, and their environments, that would otherwise be lost, to the broader scientific community [4,21]. Regular wildlife sampling and specimen archiving with biorepositories is an essential component of zoonotic pathogen detection, identification, monitoring, and mitigation, in concert with ongoing human and livestock disease monitoring [6]. The global scale of EIDs, exemplified by the current SARS-CoV-2 pandemic, calls for an intentional shift away from current reactive responses to EIDs, toward more proactive models that encourage early detection, identification, monitoring, and prevention (e.g., Documentation, Assessment, Monitoring, Action [DAMA] protocol [6,49]; Fig 1), possible by expanding One Health approaches to include the Global Museum [23–28]. To this end, we must invest in expanded biorepository capacity, quality, and expertise to meet the needs of EID research, an approach to building infrastructure that has not yet been normalized across disciplines [4–5].

### Uniting biomedicine and biorepositories in EID research and response

Although museum biorepositories exist in nearly all countries, their collections too often remain insufficient to meet the needs of EID research, including the key steps of host identification and wildlife surveillance over time and across geography. Tissue collections, in particular, remain woefully underdeveloped for most biodiverse regions of the planet, for a number of reasons. Challenges to biorepository establishment, maintenance, and growth in these regions include political turmoil, environmental conditions that challenge long-term environmental stability required for specimen maintenance (e.g., heat, humidity), and insufficient financial resources to support infrastructure, collections growth, database management, and permanent personnel [53–57]. For example, South America harbors more than double the mammal diversity of North America [58], but biorepositories in this region only house approximately 15% of all mammal specimens in the Western Hemisphere [57]. In addition, the majority of collections in South America do not yet meet gold-standard environmental conditions for long-term specimen preservation, with only 2 South American biorepositories accredited by the American Society of Mammalogists: Universidad Nacional de Tucumán, Colección de Mamiferos Lillo (CLM, Argentina) and Pontificia Universidad Católica del Ecuador, Museo de Zoología-Mamiferos (QCAZ, Ecuador) [57].

How can the scientific community initiate and establish a more integrated and effective approach to zoonotic pathogen detection, mitigation, and prevention, and ensure global adoption? Enormous value lies in leveraging the Global Museum, the existing, decentralized, global network of biorepositories, which already has collected, preserved, cataloged, and indexed tissue samples from >1 million wild vertebrate specimens worldwide (vertnet.org; accessed February 28, 2021). Strategic investment in biorepository infrastructure, including updated cryogenic tissue storage facilities, climate-controlled specimen housing, digital databases, and permanent positions for trained personnel, particularly in biodiverse countries, will be essential to building and scaling a high-quality network of global collections that meet established best practices and allow rapid response to EIDs [6,59–60]. In many biodiverse countries, biorepositories are housed at governmental institutions where infrastructure and staffing expansion may be hampered by slow, bureaucratic processes. Given the urgency of EIDs, new partnerships among institutions from different countries or with nongovernmental organizations offer new and alternative ways to grow in-country biorepository capacities more quickly. Expanded global biodiversity infrastructure is critical to ensuring sufficient biorepository capacity to stimulate, support, and respond to the growing needs of EID research as emergence frequency and severity increases.

### Project ECHO: A global communication network to unite biorepositories and biomedicine

Infrastructure alone will not ensure strategic collections growth or broad utilization of collections in EID research. Rather, meeting these goals will require new models of communication and collaboration that connect biorepositories more effectively with biomedical and public health communities [17,61]. To achieve this goal, we propose an extension of Project ECHO (Extension for Community Healthcare Outcomes [62–65]), an innovative, “community of practice” model of virtual communication, to better connect biorepositories with biomedicine across international borders. Originally developed in a clinical context, Project ECHO uses video conferences to support virtual, case-based, collaborative problem-solving, mentoring, and group learning among multidisciplinary teams of healthcare providers through nearly 1,000 programs engaging thousands of participants in >100 lower-income countries. Under this model, small groups of interested participants, geographically dispersed across countries and continents, meet regularly via video conference to share best practices across disciplines and resolve barriers through collective experience and expertise and is a proven-effective cost-saving approach to international communication and collaboration [66].

**Fig 2:**
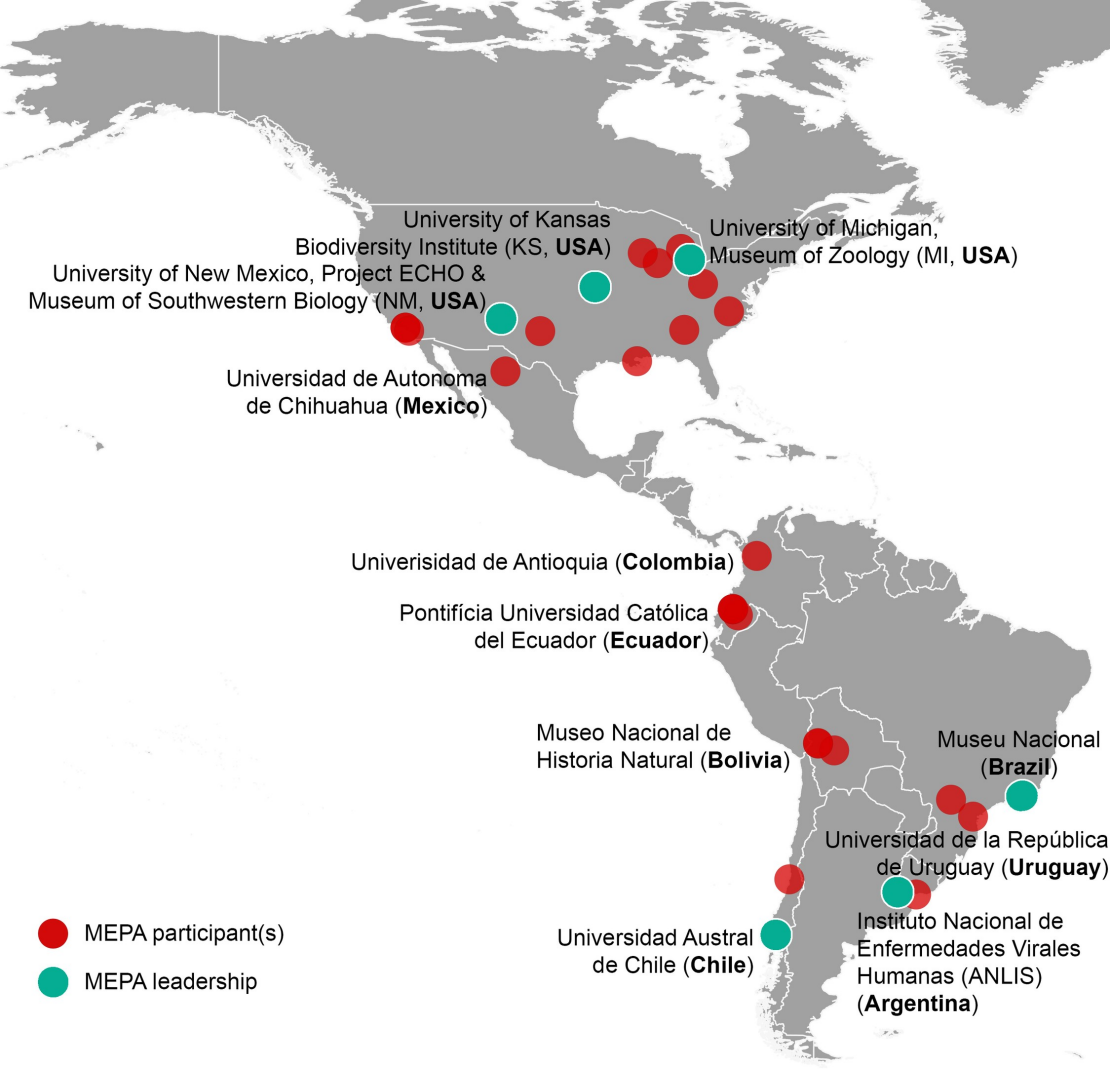
Distribution of institutions participating in MEPA as of February 2021. At least 1 participating institution is labeled per country (plus all leadership institutions). Continent base layer from Natural Earth, available online at https://www.naturalearthdata.com/downloads/10m-cultural-vectors/10m-admin-0-countries/. MEPA, Museums and Emerging Pathogens in the Americas.

Here, we highlight recent development of a regional, pilot ECHO network that aims to unite biorepositories with biomedical and public health communities across the Americas: Museums and Emerging Pathogens in the Americas (MEPA). As of April 2021, the MEPA ECHO network is composed of researchers, practitioners, policy makers, and advanced students from 9 countries in Central, North, and South America and is growing (Fig 2). MEPA aims to foster communication and coordination of zoonotic pathogen detection, host identification, and emerging pathogen research and surveillance through mutual sharing of best practices, cross-disciplinary training, and collaborative specimen sourcing. MEPA now serves as a model of international communication among biorepository and biomedical communities that can be adopted globally, regionally, or nationally. Although access to reliable internet is an anticipated barrier to this communication model, global internet connectivity is expanding, with 86% of the world population now using mobile broadband services [67]. The virtual platform also allows groups or institutions to join virtually from a single location where many individuals benefit from a single internet access point. Informal, web-based communication platforms like MEPA may further function as an early warning system for emerging diseases [68], as proved effective for SARS [69] and influenza A (H1N1, swine flu) [70].

The importance of biorepositories is acknowledged broadly in human medicine [71–72], but wildlife biorepositories remain absent from many EID research programs and, importantly, from many One Health programs, evidenced by the absence of voucher specimen collection from recent, large-scale wildlife and wildlife pathogen surveys (e.g., PREDICT/STOP Spillover [73] and NEON [74–75]). Without voucher specimens, wildlife sampling events represent missed opportunities for scientific rigor and limit the extensibility and replicability of the original science. Coordinated sampling through MEPA will promote wildlife surveys that are temporally deep, spatially extensive, and taxonomically broad so that biorepositories can be probed rapidly and effectively in the event of disease emergence to identify reservoirs and ecological and environmental associations contributing to emergence or spillover. Opening dialogues between biorepository and biomedical communities is a first step toward building new specimen-transfer pipelines, whereby specimens collected for biomedical surveillance or other initiatives are deposited in biorepositories and then loaned back to biomedical and other scientific communities for research. The MEPA network aims to connect ongoing and future biomedical wildlife surveillance with new and existing in-country biorepositories to avoid unnecessary duplication of sampling effort, by building new specimen pipelines that collectively contribute to the strategic growth of international biorepositories.

Through mutual sharing of best practices in a virtual community of practice, biorepository personnel can standardize and prioritize the types of materials and preservations most useful to EID research. Likewise, the EID community can better integrate biorepository data standards into specimen collection and sampling procedures [5,21,76–78]. Darwin Core (dwc.tdwg.org) stable identifiers, for example, represent a common vocabulary intended to facilitate the sharing of information about biological diversity and specimens [79]. Darwin Core identifiers are regularly employed by biorepositories for organizational and data management purposes, but extended use in emerging disease research has the potential to increase standardization, expedite data and specimen retrieval and integration into biorepositories, and facilitate comparisons across studies by digitally linking host–pathogen information to wildlife conservation and public health [77]. Cross-discipline communication between biorepositories and the EID community will also help normalize specimen deposition in biorepositories as an expected component of biomedical and public health workflows [4]. As a working example of this model, in 2000, the Gorgas Institute in Panama responded to a lethal outbreak of Hantavirus Pulmonary Syndrome (HPS) by contacting the Museum of Southwestern Biology (University of New Mexico) for guidance on surveying small mammals for viral pathogens. The resulting public health–biorepository collaboration leveraged multi-institutional resources and expertise to identify wildlife reservoirs of the disease quickly and provide appropriate public health guidance. The collaboration [80] resulted in >11,000 archived specimens over 2 decades, which now serve as a foundation for continued monitoring of and research into hantaviruses and their hosts, in addition to other symbionts and pathogens. Similar responses to HPS emergence in Chile and Argentina spawned parallel specimen-based research efforts that identified new hantavirus strains in a number of rodent species [81–83], underscoring the power of public health–biorepository collaborations to build comprehensive biological archives useful for EID research and response. Fundação Oswaldo Cruz (Fiocruz, Brazil) provides another precedent of successful biorepository and public health integration to maximize EID research and response. Fiocruz is contracted by the Brazilian Department of Health to respond to disease outbreaks by sampling and archiving potential reservoirs. Samples are then available to the EID research community and also used in ecological, evolutionary, and environmental investigations. Fiocruz also offers a taxonomy reference service to ensure accurate host identification. MEPA uses case-studies like these to guide formation of effective new collaborations between biorepositories and public health. Several other international initiatives, like the Global Taxonomy Initiative formulated by the Convention on Biological Diversity and the Belgian Development Cooperation, have also made steps to increase access to best practices, although enormous knowledge and resource gaps remain [84–88].

Preservation of specimens collected for EID research and surveillance is expected to massively increase the volume of archived materials. With expanded infrastructure and support for trained personnel, as outlined above, biorepositories will be well equipped to meet this challenge, as they already have streamlined accession and loan procedures, international import/export permits, and the wet lab and, in many cases, appropriate BioSafety Level certifications to safely process large volumes of biological material. A lack of funds, training, and support for permanent personnel has hindered biorepository productivity and capacity, particularly in biodiverse, lower- to middle-income countries where basic taxonomy and baseline conditions too often remain uncharacterized [89,90]. In response, biodiversity literacy and training form another core mission of MEPA. The Biodiversity Informatics Training Curriculum [91] provides a mixture of in-person courses across Africa and online courses and resources served via YouTube (332,000+ views and 45,000+ hours of viewing to date) to leverage natural history collections data [91] and provides a model of a digital format, open-access training tool for MEPA to build from. Advancing Integration of Museums into Undergraduate Programs (AIM-UP! [92]), Biological Collections in Ecology and Evolution Network (bceenetwork.org), and Biodiversity Literacy in Undergraduate Education (BLUE [93]) are similar digital learning tools that can be expanded and served to the international biodiversity community to build a global workforce capable of developing, organizing, and managing biological materials and data, but also able to wield the latest technologies to connect biorepository resources to issues of societal concern. Hybrid training programs that integrate medical (human and veterinary) and public health expertise with biodiversity science are urgently needed to improve the translation of natural history research into actionable policy [6,94–96].

### Moving forward: Stakeholder recommendations for building better biorepositories

**Fig 3:**
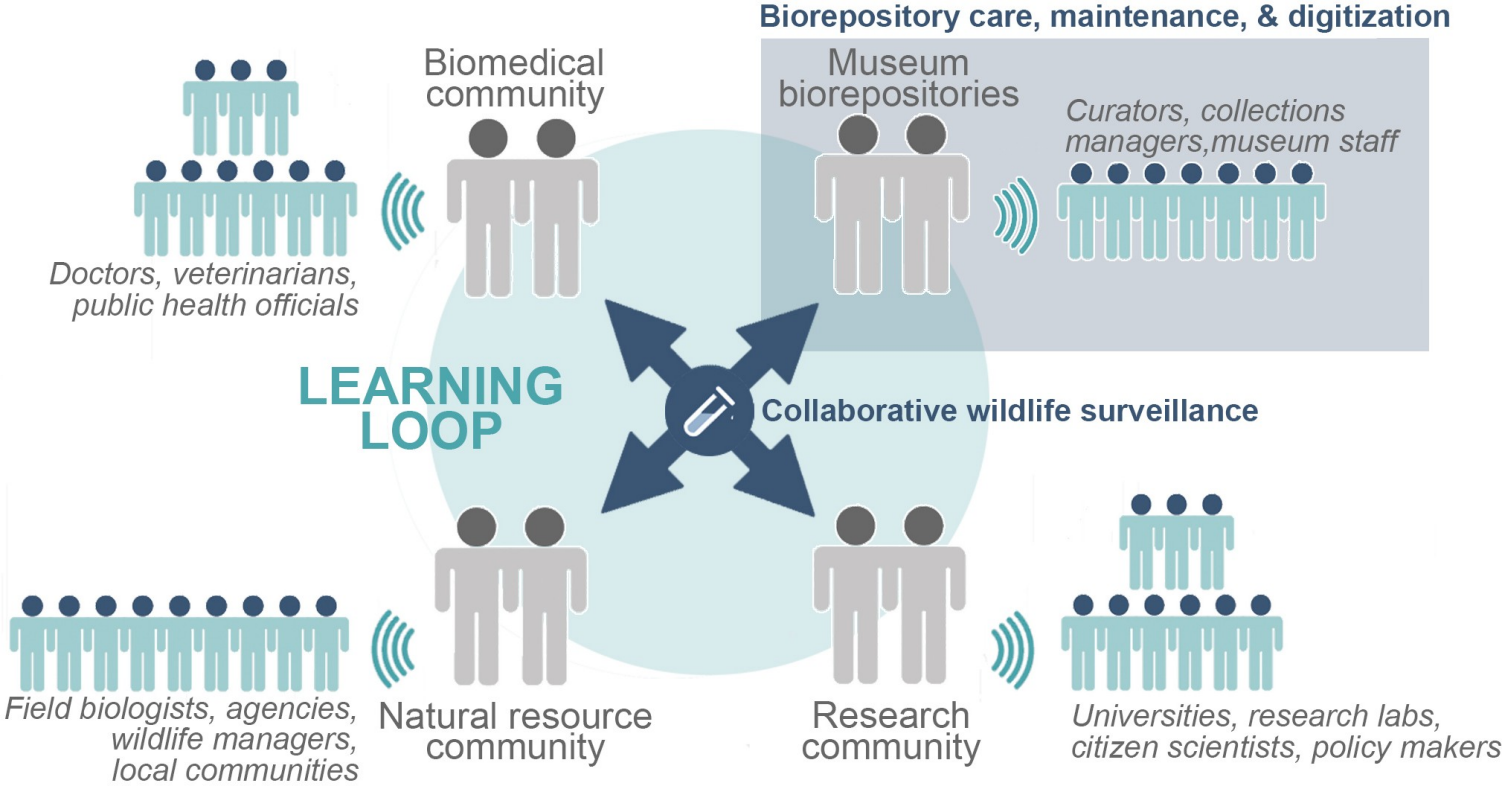
Multi-stakeholder Project ECHO learning loop or “hub” model that unites biorepository (e.g., museum), biomedical, natural resource, and research (e.g., EID, biodiversity, ecology, evolution, virology, pathobiology, etc.) communities, among others, to reciprocally share best practices and develop global wildlife sampling strategies for effective EID research and response, as well as studies of biodiversity. Under this model, geographically and taxonomically diverse specimens collected across disciplines and projects will be permanently archived in biorepositories for **(a)** long-term care, maintenance, and digitization, and **(b)** continued use and reuse by the global research community. EID, emerging infectious disease; ECHO, Extension for Community Healthcare Outcomes.

Together, expanded global biorepository infrastructure and new communication pipelines can help prevent future zoonotic pandemics before they occur [49] and reinforce efforts to mitigate EIDs, as recommended by current One Health approaches. There are long-term, highly successful examples of international collaborations between biomedical and biorepository communities (e.g., the Gorgas Institute-Panama and Museum of Southwestern Biology-USA; Fiocruz and National Museum of Brazil) that serve as models for building new collaborative relationships. EID research and response are inherently multidisciplinary endeavors; thus, we encourage all stakeholders (public health officials, curators, veterinarians, doctors, epidemiologists, virologists, parasitologists, basic and clinical research scientists, wildlife managers, field station managers, natural resource managers, funding agencies, and policy makers, among others) to contribute to the collective growth and availability of wildlife samples and associated informatics through open-access biorepositories (Fig 3) [97]. Local buy-in for field-based activities and collections and incorporation of perspectives based on traditional ecological knowledge [10,98] are also essential in the feedback process that links basic biodiversity research about hosts and pathogens to actionable processes for people at local and regional levels. To this end, we have identified a series of priority actions for the diverse stakeholders in this network.

#### Biorepositories

Biorepositories (e.g., natural history museums or biobanks) must actively align their long-term archives with issues of societal concern, which often revolve around changing conditions (e.g., climate warming, globalization, emerging pathogens, food security, and invasive species). Biorepositories cannot afford to serve only a limited set of users; instead, they must embrace new specimen sources and formats, as well as new technologies and applications [21,99,100]. As trusted sources of biodiversity information and science, biorepositories are well positioned to unify existing stakeholders in EID research, preparedness, and response and communicate scientific results and discoveries that bridge humans, wildlife, and the environment to policy makers and the public.

To this end, specimens and their associated data must be made readily available to the entire scientific community [4] and most importantly, to the countries from where the specimens originate [13,14,101]. The Nagoya Protocol is an international agreement designed to foster the sharing of benefits arising from the utilization of genetic resources in a fair and equitable manner [102–106]. Equitable benefits sharing is essential to successful integration of biomedical and biorepository communities; however, despite its intention, the Nagoya Protocol has had a number of unintended negative consequences on international biodiversity research, making it increasingly difficult for researchers and scientists to access and share specimens across borders [103]. Historically, legislation surrounding benefits sharing and the use of genetic resources pertained primarily to commercial profits and products, but the concept of benefits sharing also includes nonmonetary products (e.g., royalties, publications, and data) and should be further expanded to include reciprocal support for in-country infrastructure development (e.g., gold-standard liquid nitrogen tissue cryobanks) and trained personnel, essential to capacity building in biodiverse countries.

The scale of EID research and monitoring proposed herein also critically relies on open access and sharing of biodiversity data, and especially genetic sequence data (GSD). Biodiversity data portals have been implemented at very large scales with >225 million specimens (approximately 31 million of which belong to Chordata) digitized worldwide (gbif.org, accessed January 5, 2021), but these data must be quality checked and updated regularly and enriched via standardization of key fields, such as reproductive status, age, sex, geographic coordinates, etc.. Trained personnel are essential to ensuring databases are kept accurate and up to date. Examples of digital biodiversity databases include the Global Biodiversity Information Facility (GBIF; gbif.org), VertNet (vertnet.org), iDigBio (idigbio.org), the Sistema de Inforcação Sobre a Biodiversidade Brasileira (SiBBR; sibbr.gov.br), the Global Genome Biodiversity Network (GGBN; ggbn.org), and speciesLink (splink.cria.org.br). Other examples, such as BioWeb (bioweb.bio), the largest biodiversity data repository in Ecuador, and the Arctos museum database (arctos.database.museum), a digital database that publicly serves data from 37 biorepositories across 3 countries, are useful examples of existing biodiversity databases available for new and growing biorepositories to join. Biorepository databases must be research grade quality, that is, openly available, based on standardized vocabularies [79], and interoperable with other large data streams (e.g., GenBank, GIS platforms, and IsoBank [21,77,107,108]). Dunnum and colleagues [57] and iDigBio (idigbio.org/content/dna-banks-and-genetic-resources-repositories-united-states) have published lists of biorepositories in the Western Hemisphere and the United States, respectively, but similar lists are critically needed for other geographic regions to encourage specimen use and deposition.

Best practices for biorepository development for pathogen exploration exist [15,60,76] and provide a guide for additional infrastructure development [109]. Collaboration with the EID community requires biorepositories to reinvest in student training and capacity-building missions, especially in biodiverse and lower-income countries, ideally with reinvigorated public investment, to ensure the next generation of biodiversity scientists are trained in skills that bridge the 2 communities. Future generations must have the ability to identify and access key samples, use the materials and data in biorepositories via the latest technologies, and fully engage in initiatives related to human and ecosystem health. Construction of in-country cryogenic tissue storage facilities, as a major component of updated biorepository infrastructure, complements student training in molecular sciences, provides access to primary scientific resources, and provides the basic infrastructure required for improved, rapid response to EIDs in addition to the regular collection, preservation, and study of baseline material. Although some biorepositories in the Global North have histories of colonial collection practices [13,110–111], MEPA focuses on capacity building in the Global South, with expanded investment in international biodiversity training [112] and sharing of existing and refined best practices [5,60,76,109] with the goal of building a more inclusive, diverse, and effective global network of biodiversity science.

#### Field biologists

Holistic collection of high-quality, information-rich specimen materials, suitable for pathogen research [15,109,113], and regular specimen archiving within biorepositories must become a normalized, standard practice across all disciplines [4–5,21]. If holistic sampling is not possible (e.g., due to logistical or conservation reasons), voucher specimens should be collected for representatives of each sex and species for each site surveyed. Sampling must be strategically distributed across taxonomic diversity and temporal and spatial scales to sample mosaic environments, at multiple ecosystem interfaces (including urban and rural environments) to strengthen the ability of biorepositories to identify and monitor potential zoonotic reservoirs over space and time (Fig 1; see DAMA protocol [49]. In addition to sampling hosts with a high incidence of zoonotic spillover (e.g., bats and rats), wildlife sampling should also include broad geographic and taxonomic surveys at a range of environmental interfaces, critical to establishing a temporal record of pathogen presence/absence and prevalence [19,109,114]. Historic collections may then be leveraged as baselines for longitudinal assessments of pathogen dynamics through time, such that resurveys of long-term sampling sites may be especially powerful. MEPA aims to connect field biologists with biorepositories to ensure responsible specimen deposition, data standardization, and access for EID and other research interests.

#### Natural resource agencies

By leveraging logistical resources and field networks, protected reserves, park and forest administrators, field stations, and wildlife management and development agencies can contribute significantly to biorepository growth by facilitating the collection and preservation of diverse samples over time and space (Fig 3) [4,115,116]. Many remote and protected areas are under the jurisdiction of natural resource agencies and represent some of the last environments not heavily impacted by humans. As such, these areas provide important baseline information on pathogens and their natural reservoirs that provides a reference for understanding disturbed and human-modified landscapes. Collections made by natural resource agencies should be archived permanently with a biorepository, not housed under potentially unsuitable environmental conditions at field stations or in lab freezers, to avoid specimen degradation and data dissociation over time, and also to ensure that resources for specimen maintenance, curation, and data digitization are available. MEPA and related ECHO networks aim to connect natural resource agencies with local biorepositories to facilitate long-term specimen preservation under ideal environmental conditions and in accessible formats. Specimen access through biorepositories maximizes the scientific impact of each sampling event, which can increase the amount of information available to decision-makers. We recommend that natural resource agencies, especially those involved in permitting collections-based research or seizure of illegally trafficked wildlife, establish collaborations with local biorepositories to regularly archive specimens, ideally as part of the permitting process [4,117].

#### Research scientists

To democratize science and enable transdisciplinary approaches to EID research and response, biological materials collected by researchers, regardless of the investigator’s discipline, motivation for collection, or affiliation, must eventually be archived permanently in a biorepository [5,118]. Research scientists should plan and budget for specimen (e.g., skin, skull, skeleton, tissues, parasites, etc.) and data deposition prior to collection as a routine step toward repeatable and responsible science [4,21]. Stable identifiers [108] are crucial to identifying specimens uniquely and can be arranged in advance with the receiving biorepository [4,21]. Researchers can connect with local biorepositories through the MEPA network, other virtual communication networks (e.g., regional ECHOs), or by reaching out to collections staff directly. Further, MEPA provides a platform for communicating new discoveries regarding EIDs and wildlife sampling to multi-stakeholder groups, including policy makers in public health and wildlife managers, which may expedite public health response [69–70].

#### Medical professionals and public health officials

Wildlife sampling motivated by public health interests must involve intentional specimen vouchering and archival with permanent and public biorepositories as a best practice. Biorepositories are reciprocally available to medical professionals and public health officials as foundational resources to draw on for rapid response to emerging zoonoses. Government-funded disease surveillance programs have ethical and fiduciary duties to voucher collected specimens appropriately, as this best practice has clear public health benefits [3–5]. Coordinating sampling of vertebrate reservoirs with permanent biorepositories to build rigorous infrastructure over time will ensure a foundation for future pandemic preparedness and investigations of pathogen maintenance and transmission, ecology and evolution, environmental associations, and prevalence, among other topics of direct interest to public health [3,19,22]. MEPA and other such communication networks provide a platform for building such cross-disciplinary connections.

#### Funding agencies and foundations

EIDs represent a major threat to global biosecurity and have cascading negative impacts on society, as illustrated by the SARS-CoV-2 pandemic. In response, national science foundations [21], international development agencies (e.g., USAID, Department for International Development [DFID] Brazil), national security organizations, private philanthropy, and the United Nations represent potential platforms for promoting large-scale international, in-country biorepository infrastructure expansion. Reframing research funding initiatives to prioritize long-term impacts, rather than immediate or short-term (3 to 4 years) responses to emerging threats or short-term research projects, will substantially increase ability to proactively address emerging pathogens. By inviting members of these funding organizations to actively participate in MEPA problem-solving sessions, we aim to increase awareness of the role of biorepositories in societal issues and identify and apply for novel funding opportunities to grow international biorepository infrastructure and biomedical capacities.

## Conclusions

The need for in-country biorepository infrastructure development and international communication channels is not limited to the Americas; the MEPA ECHO network is a pilot project, modeling effective interdisciplinary communication and collaboration across international borders that can and should be replicated and expanded to include other corners of the globe such as biodiversity hotspots in Africa and East and Southeast Asia. Emulating Project ECHO’s “hub and spoke” model, we envision multiple regional ECHO networks bridging biorepositories and biomedicine that meet regularly to coordinate wildlife surveillance, communicate new discoveries, and standardize sample collection and preservation procedures. Regional networks would then periodically connect with related biodiversity-based ECHO networks at a global level for meta-regional meetings coordinated by MEPA. Meta-regional meetings will feature a brief roundtable of intercontinental updates and focus collectively on problem-solving current issues in emerging disease.

Biorepositories are the primary biodiversity infrastructure of the planet and form the foundation of the wildlife branch of a One Health (human–wildlife–environment) approach to EIDs. Although traditionally funded through basic science, the biorepository funding base must expand to include funding sources that support research into scientific issues of direct benefit to human society (e.g., accelerating climate warming, EIDs, food security, globalization, biodiversity loss, and invasive species). Costs of new and expanded biodiversity infrastructure, including updated cryogenic facilities and support for trained personnel, and new communication channels are minimal when weighed against the impact of lives lost, economies disrupted, and trillions of dollars spent combating EIDs (e.g., the >$20 trillion US dollars spent in the first year of the SARS-CoV-2 pandemic in the US [119]; $21 to $31 trillion spent in the global arena [120]). Expanded in-country biodiversity infrastructure and open channels of communication that foster interchange and collaboration among the Global Museum and biomedical communities offer an opportunity to leverage ongoing wildlife surveillance to begin a transformation of EID research into an increasingly proactive and predictive science to enable rapid public health response and reduce both the chances and costs of future outbreaks.
